# Diaphragmatic Excursion as a Novel Objective Measure of Serratus Anterior Plane Block Efficacy: A Case Series

**DOI:** 10.5811/cpcem.2022.7.57457

**Published:** 2022-11-04

**Authors:** Brian Lentz, Sigmund Kharasch, Andrew J. Goldsmith, Joseph Brown, Nicole M. Duggan, Arun Nagdev

**Affiliations:** * Highland Hospital-Alameda Health System, Department of Emergency Medicine, Oakland, California; † Massachusetts General Hospital, Department of Emergency Medicine, Boston, Massachusetts; ‡ Brigham and Women’s Hospital, Department of Emergency Medicine, Boston, Massachusetts; § University of Colorado, Department of Emergency Medicine, Aurora, Colorado

**Keywords:** ultrasound, diaphragmatic excursion, nerve block, pain scale, case report

## Abstract

**Introduction:**

Pain scales are often used in peripheral nerve block studies but are problematic due to their subjective nature. Ultrasound-measured diaphragmatic excursion is an easily learned technique that could provide a much-needed objective measure of pain control over time with serial measurements.

**Case Series:**

We describe three cases where diaphragmatic excursion was used as an objective measure of decreased pain and improved respiratory function after serratus anterior plane block in emergency department patients with anterior or lateral rib fractures.

**Conclusion:**

Diaphragmatic excursion may be an ideal alternative to pain scores to evaluate serratus anterior plane block efficacy. More data will be needed to determine whether this technique can be applied to other ultrasound-guided nerve blocks.

## INTRODUCTION

Peripheral nerve blocks are an important component of multimodal analgesia for thoracic pain.^1^ The serratus anterior plane block (SAPB) involves placing local anesthetic into the fascial plane between the serratus anterior and latissimus dorsi muscles, or between serratus anterior and an underlying rib, using real-time ultrasound guidance.^2^ Serratus anterior plane block can be used in a variety of settings including after surgery involving the chest wall or in the emergency care setting for anterior and/or lateral rib fractures. Rib fractures occur in 9–10% of all trauma patients. Controlling chest wall pain in these patients is crucial as inadequately treated pain is associated with increased risk of chest wall splinting leading to hypoventilation, atelectasis and, eventually, pneumonia.^3^,^4^ Diaphragmatic excursion has been proposed as a surrogate objective method of respiratory status in this patient subpopulation.^5^

Point-of-care ultrasound (POCUS) evaluation of diaphragmatic excursion can provide the quantification of diaphragmatic function over time through serial evaluation, and it has high sensitivity and specificity compared to chest radiography.^5^–^7^ Further, diaphragmatic dysfunction can be caused by a variety of interventions and diseases including mechanical ventilation, cardiac and abdominal surgery, phrenic nerve injury, neuromuscular disorders, lung hyperinflation, and multi-organ dysfunction in critical illness.^8^ Several reviews describing POCUS uses and techniques to evaluate the diaphragm have been published.^9^–^11^

Specifically, diaphragmatic excursion may provide a quantification method of respiratory status after intervention. As visual pain scores are a subjective perception of an individual’s pain, objectively comparing this measurement has been a challenge in peripheral nerve block studies.^12^ The excursion of the dome of the diaphragm can be used to guide clinicians on the degree of respiratory compromise in specific pulmonary pathologies.^13^,^14^ As splinting secondary to rib fractures is a known phenomenon, diaphragmatic ultrasound may provide an objective outcome of successful nerve blocks for rib fractures. We propose diaphragmatic excursion as a new objective outcome of block efficacy in thoracic nerve blocks. Here we describe three cases where diaphragmatic excursion was used as an objective measure of SAPB efficacy in emergency department (ED) patients with anterior or lateral rib fractures.

**Image 1:**
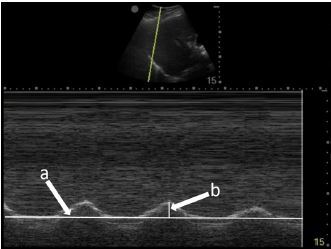
Diaphragmatic excursion is calculated by first determining a baseline (line a) and then measuring the distance of maximal vertical excursion (distance b).

To measure diaphragmatic excursion the patient is first placed in a supine position. A curvilinear probe is placed in the midaxillary line and oriented cephalad to optimally visualize the inferior aspect of the lungs, diaphragm, and upper abdomen (ie, spleen or liver). Diaphragmatic excursion was quantified on M-mode imaging, with the M-mode cursor directed through the diaphragm. The amplitude of diaphragmatic excursion was measured from the baseline to the point of maximal excursion on the vertical axis (Image 1).

Nagdev et al provide a complete description of SAPB.^15^ In summary, the patient is placed in a lateral decubitus or supine position. A high-frequency linear transducer is placed in the midaxillary line at the level of the nipple to locate the serratus anterior muscle. A blunt-tip block needle is then used to inject anesthetic into the plane between the serratus anterior muscle and latissimus dorsi muscles. To perform the block, the needle is visualized using an in-plane approach until the tip is located just above the serratus anterior muscle. Once the correct position is confirmed, a large volume of anesthetic is injected into the fascial layer. As with all blocks, intralipid should be readily available in the event of local-anesthetic systemic toxicity syndrome.

**CPC-EM Capsule**

What do we already know about this clinical entity?

*Pain scales are often used to measure the efficacy of peripheral nerve blocks but are problematic due to their subjective nature*.

What makes this presentation of disease reportable?

*This is the first description of diaphragmatic excursion as an objective measure of appropriate pain control in acute rib fractures in the emergency care setting*.

What is the major learning point?

*Diaphragmatic excursion is a promising novel tool to quantify improved pain and respiratory function after serratus anterior nerve block and possibly other blocks*.

How might this improve emergency medicine practice?

*This technique could be utilized in future studies of nerve block efficacy as well as clinically to guide appropriate pain control*.

## CASE SERIES

### Case 1

**Image 2:**
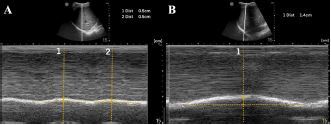
(A) Pre-block demonstrating right-sided diaphragmatic excursion of 5 millimeters (mm) (average of two excursions). (B) Post-block demonstrating right-sided diaphragmatic excursion of 14 mm (increase of 64%).

A 44-year-old man presented after a motorcycle collision and was found to have right-sided fractures of ribs 2–5 and 12 on computed tomography (CT). The patient continued to report severe pain after 100 micrograms (mcg) of intravenous (IV) fentanyl. M-mode of the right diaphragm was performed prior to SAPB and showed 5 millimeters (mm) of diaphragmatic excursion and a respiratory rate of 24 breaths per minute (BPM) (Image 2A). A right ultrasound-guided SAPB was performed with 20 milliliters (mL) of 1% ropivacaine. On re-evaluation approximately 60 minutes later, M-mode of the right diaphragm showed a respiratory rate of 16 BPM and 14 mm of diaphragmatic excursion (increase of 64%) (Image 2B). Increase in diaphragmatic excursion was calculated as the change in diaphragmatic excursion (14 mm minus 5 mm) divided by the post-block diaphragmatic excursion (14 mm).

### Case 2

**Image 3:**
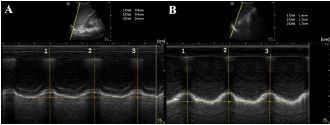
(A) Pre-block demonstrating left-sided diaphragmatic excursion of 8 mm (average of three excursions). (B) Post-block demonstrating left-sided diaphragmatic excursion of 17 mm (increase of 53%).

A 35-year-old man presented after an assault and was found to have a left lateral sixth rib fracture on CT. The patient received 1000 milligrams (mg) of IV acetaminophen but continued to report severe pain. M-mode of the left diaphragm was performed prior to SAPB and showed 8 mm of diaphragmatic excursion and a respiratory rate of 20 BPM (Image 3A). A left ultrasound-guided SAPB was performed with 20 mL of 1% ropivacaine. On re-evaluation approximately 60 minutes later, M-mode of the left diaphragm showed 17 mm of diaphragmatic excursion (increase of 53%) and a respiratory rate of 16 BPM (Image 3B).

### Case 3

**Image 4:**
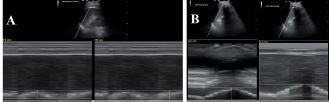
(A) Pre-block demonstrating left-sided diaphragmatic excursion of 19 mm. (B) Post-block demonstrating left-sided diaphragmatic excursion of 32 mm (increase of 41%).

A 50-year-old man presented with left-sided chest wall pain after a fall four days prior when intoxicated and was found to have fractures of ribs 6–9 on CT. The patient initially rated his pain 10/10 and was given 100 mcg of IV fentanyl. Pain continued to be 10/10 and a SAPB was performed for pain control. A left ultrasound-guided SAPB was performed with 20 mL of 0.5% bupivacaine combined with 10 mg of dexamethasone. The patient’s pain 60 minutes after the block was 2/10. A diaphragmatic POCUS was performed both before and 60 minutes after the SAPB block. The initial respiratory rate was 20 BPM with 19 mm of diaphragmatic excursion (Image 4A). After 60 minutes from the SAPB, the patient’s respiratory rate was 14 BPM with a diaphragmatic excursion of 32 mm (increase of 41%) (Image 4B).

All blocks were performed by fellowship-trained ultrasound faculty. The vital signs of all the patients were stable; specifically, no patient had hypotension or hypoxia prior to the block being performed. No additional pain medications were given prior to the second diaphragmatic excursion exam.

## DISCUSSION

To our knowledge this is the first description of using diaphragmatic excursion as a measurement of appropriate pain control in acute rib fractures. In our cases, after SAPB the respiratory rate decreased while M-mode measured diaphragmatic excursion increased. This initial data may suggest a new objective measure of improved pain control for acute rib fractures as well as decreased respiratory splinting. Ultrasound-measured diaphragmatic excursion may provide a much-needed objective measure of respiratory improvement in acute rib fracture, since subjective pain scores are typically the only measures used during clinical care.^12^

Diaphragmatic excursion may also be used as a signal for overall pain. Pain can be evident in some patient populations by an increase in respiratory rate and more shallow breaths for all acute painful conditions. Diaphragmatic excursion may directly demonstrate improved pain control and respiratory function following ultrasound-guided peripheral nerve blocks, and not just for thoracic trauma as shown in these three cases. Although subjective pain scales have a role in measuring block effectiveness, they are an indirect measure of respiratory function and problematic in cases where there are distracting injuries or altered mental status. Diaphragmatic excursion, however, can be used in all patients and could define that ultrasound-guided nerve blocks both relieve pain in the form of decreased splinting as well as directly improve pulmonary function. This technique has the potential to be valuable both in future studies of nerve block effectiveness and to help guide adequate pain control in clinical situations where patients are not able to communicate subjective pain using visual pain scores.

The POCUS-measured diaphragmatic excursion is both easily learned and non-invasive, making it an ideal objective measure. Although this case series demonstrates diaphragmatic excursion as a promising novel tool to quantify pain and respiratory splinting, it has several limitations. Data regarding interrater reliability was not available as diaphragmatic measurements were obtained by a single clinician pre- and post-block. Although a standardized technique was used to measure diaphragmatic excursion, a possibility of measurement error exists from differences in probe placement pre- and post-block. Lastly, subjective pain scores pre- and post-block were only available for the third case, and spirometry data was not collected for comparison. Point-of-care ultrasound measured diaphragmatic excursion is both easily learned and non-invasive, making it an ideal objective measure.

## CONCLUSION

Serratus anterior plane bock performed for acute rib fractures reduced pain in all three patients. It also increased the diaphragmatic excursion and decreased the respiratory rate in all three cases. Diaphragmatic excursion may be an alternative to visual pain scores to evaluate SAPB efficacy. More data will be needed to determine whether this same relationship can extend to other ultrasound-guided nerve blocks.
